# Ethylenediamine-*N*,*N*′-Disuccinic Acid (EDDS)—Enhanced Flushing Optimization for Contaminated Agricultural Soil Remediation and Assessment of Prospective Cu and Zn Transport

**DOI:** 10.3390/ijerph15030543

**Published:** 2018-03-18

**Authors:** Marco Race, Alberto Ferraro, Massimiliano Fabbricino, Agostino La Marca, Antonio Panico, Danilo Spasiano, Alice Tognacchini, Francesco Pirozzi

**Affiliations:** 1 Department of Civil, Architectural and Environmental Engineering, University of Naples Federico II, via Claudio 21, 80125 Naples, Italy; massimiliano.fabbricino@unina.it (M.F.); agostino.lamarca@libero.it (A.L.M.); francesco.pirozzi@unina.it (F.P.); 2 Department of Civil and Mechanical Engineering, University of Cassino and Southern Lazio, 03043 Cassino, Italy; alberto.ferraro3@gmail.com; 3 Telematic University Pegaso, Piazza Trieste e Trento 48, 80132 Naples, Italy; antonio.panico@unipegaso.it; 4 Department of Environmental, Building, Civil Engineering and Chemistry, Polytechnic University of Bari, Via Orabona n.4, 70126 Bari, Italy; danilo.spasiano@poliba.it; 5 Department of Forest and Soil Sciences, University of Natural Resources and Life Sciences, Vienna, Institute of Soil Research, Konrad-Lorenz-Straße 24, 3430 Tulln, Austria; alice.tognacchini@yahoo.it; 6 Alchemia-nova GmbH, Baumgartenstrasse 93, 1140 Vienna, Austria

**Keywords:** metal mobility, soil flushing, soil reclamation, agricultural soil, EDDS deficiency

## Abstract

This paper presents the results of an experimental study aimed at investigating the effect of operative parameters on the efficiency of a soil flushing process, conducted on real contaminated soil containing high amounts of Cu and Zn. Soil flushing tests were carried out with Ethylenediamine-*N*,*N*′-disuccinic acid (EDDS) as a flushing agent due to its high biodegradability and environmentally friendly characteristics. Process parameters such as Empty-Bed Contact Time (EBCT) and EDDS solution molarity were varied from 21–33 h and from 0.36–3.6 mM, respectively. Effects on the mobility of cations such as Fe and Mn were also investigated. Results showed that very high performances can be obtained at [EDDS] = 3.6 mM and EBCT = 33 h. In these conditions, in fact, the amount of removed Cu was 53%, and the amount of removed Zn was 46%. Metal distribution at different depths from the top surface revealed that Cu has higher mobility than Zn. The process results were strongly dependent on the exchange of metals due to the different stability constants of the EDDS complexes. Finally, results from a comparative study showed that soil washing treatment reached the same removal efficiency of the flushing process in a shorter time but required a larger amount of the EDDS solution.

## 1. Introduction

Illegal disposal of industrial waste containing different pollutants, both organic and inorganic, is responsible for severe agricultural soil pollution [1]. Although such contamination can be related to the presence of so-called emerging pollutants, such as liquid-ionic pollutants or pesticides [2], in most cases it is related to the presence of potentially toxic elements (PTEs) [3,4]. Among others, the presence of Cu and Zn is quite common, because these metals have a wide application in numerous industrial processes as well as in the production of many pesticides and herbicides [5]. The remediation of PTEs-contaminated soils can be carried out using different techniques. Among them, the soil washing process proved to be one of the few permanent contaminated soil remediation treatments [6]. Therefore, this process can achieve good remediation performances if the operative conditions are carefully selected and optimized [7,8,9]. However, in the case of soil washing treatment, the excavation of the contaminated soil is required, and this operation is time-consuming and expensive. This step is then followed by an in-site or on-site treatment by mixing the solid phase with a washing solution able to solubilize the pollutants in the liquid phase. The two phases are successively separated. The remediated soil can be returned to its original place [10] or used for different purposes. The contaminated washing solution, on the other hand, must be appropriately treated [8,11,12] before its final disposal. Unfortunately, this process cannot be applied if the contamination is extended to large or deep areas, because of the excessive costs related to the soil excavation. In these cases, an interesting alternative to soil washing is soil flushing [13,14]. Soil flushing consists of the direct injection in the soil of a leaching solution, avoiding the necessity of contaminated soil excavation. 

Several chemical reagents have been positively tested in the past as leaching solutions for soil remediation in the flushing process (i.e., HCl, EDTA, CaCl_2_) [15,16]. However, part of the flushing solution can be retained in the soil and/or leaches into the groundwater, representing a further source of environmental contamination. Therefore the use of a biodegradable agent, such as Ethylenediamine-*N*,*N*′-disuccinic acid (EDDS) [17,18], may be more advisable in order to decrease the potential risk of negative effects on the environmental quality. While the application of EDDS as a washing agent has been widely studied in several scientific research papers [9,19,20], its use as flushing agent has been tested in limited cases [21,22], and thus requires further investigation in more detail. 

The present paper aims to provide a deeper understanding of the applicability of EDDS-enhanced flushing for the remediation of Cu- and Zn-contaminated agricultural soil. The study was conducted at the lab scale to keep all process parameters under control, as well as to analyze the removal efficiency and the extraction kinetic trend. Metals distribution at different depths was also investigated to understand the mechanisms of metal desorption and adsorption during transportation, according to their initial distribution in the different contaminated soil fractions. Moreover, the release of PTEs in the leachate after the soil flushing treatment was evaluated. Finally, results from EDDS-enhanced washing tests conducted at the lab scale on the same soil were reported in order to compare the pollutants removal efficiency and the treatment environmental suitability of the two processes (i.e., soil flushing and soil washing). All of these aspects have been rarely considered in a single experimental work, thus reflecting the novelty of this study.

## 2. Materials and Methods

### 2.1. Reagents and Analytical Standards

Hydroxylammonium chloride (reagent grade >98% *w*/*w*), ammonium acetate (>99% *w*/*w*), (S,S)-ethylenediamine-*N*,*N*′-disuccinic acid-trisodium salt solution (35% *v*/*v*), hydrogen peroxide solution (30% *v*/*v*), acetic acid (ACS reagent >97% *v*/*v*), and nitric acid (ACS reagent >67% *v*/*v*) from Sigma-Aldrich (Saint Louis, MI, USA) were used as reagents. Only ultra-pure water was used for analytical preparations and dilution. Atomic Absorption Spectrometer (AAS) standards were employed for Cd, Cr, Cu, Fe, Pb, Zn (Carlo Erba, Reagenti), Mn, and Ni (Fluka Reagents).

### 2.2. Soil Characterization

**Figure 1:**
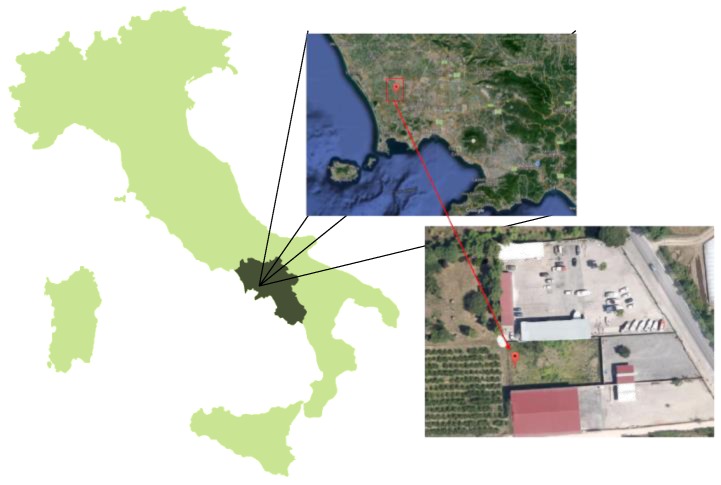
The sampling point (40°96′05″ N, 14°11′84″ E).

The investigated soil was sampled from a land used in the past for agriculture and located in the Litorale Domizio Flegreo and Agro Aversano (Campania Region, Italy) (Figure 1). In particular, this land includes an area of 1076 km^2^ (57 municipalities) and has been affected by the illegal disposal of hazardous wastes [23,24]. The soil was sampled manually from the top 20 cm of the soil over an area of about 1 m^2^, homogenized, and then stored in hermetic containers. Immediately after collection, the samples were dried at 40 °C in an oven (Argolab, TCN115) and then kept at room temperature. 

The particle size distribution analysis was performed according to American Society for Testing and Materials (ASTM) method D 422-63 [25]. Only the fraction of soil that was smaller than 2 mm, which was assumed to be the most contaminated [26], was used in all of the tests and analytical determinations. The pH of the soil was evaluated according to the method of Violante ed Adamo [27]. Organic matter was determined through the loss on ignition (LOI) index [28]. Total PTEs concentration was measured in the liquid phase from soil acid mineralization procedure [29]. Before the analysis, the solid and liquid phases from the acid mineralization were separated through centrifugation at 4600 rpm for 20 min using an IEC CENTRA GP8R centrifuge (Needham Heights, MA, USA). Then, the resulting liquid phase was analyzed by atomic adsorption spectrometry using a Varian spectrometer, Model 55 B SpectrAA (F-AAS) (Varian Australia Pty Ltd., Victoria, Australia) equipped with a flame (acetylene/air) and a deuterium background correction. The limit of detection (LOD) values for each of the analyzed elements were 5 × 10^−2^ mg·L^−1^ for Cd and Zn, 0.5 mg·L^−1^ for Cr, 0.2 mg·L^−1^ for Cu, 0.25 mg·L^−1^ for Fe, 0.1 mg·L^−1^ for Mn, 0.3 mg·L^−1^ for Ni, and 1 mg·L^−1^ for Pb. A sequential extraction procedure proposed by the Community Bureau of Reference (BCR) [30,31] was performed on the contaminated soil samples to determine the metal partition among different soil fractions. This procedure was based on an initial extraction in 40 mL of acetic acid (0.11 M) (step 1—exchangeable and weak acid soluble fraction). Afterward, a volume of 40 mL of hydroxylammonium chloride solution (0.5 M) was added to the residual soil from “step 1” and acidified by the addition of a 2 M HNO_3_ solution (step 2—reducible fraction). Successively, 20 mL of hydrogen peroxide (8.8 M) and 50 mL of ammonium acetate (1 M) were used for oxidizing the soil (step 3—oxidizable fraction). The last step consisted in a soil acid mineralization (step 4—residual fraction).

### 2.3. Soil Flushing Lab Scale Tests

Soil flushing tests were performed following the indication of Hauser et al. [21]. In particular, the columns were prepared using two polypropylene “falcon” test tubes characterized by a diameter and length of 30 and 80 mm, respectively. Tests were carried out by filling each tube with 40 g of the investigated soil. Soil was packed in the columns until no reduction of volume was observed, according to Pontoni et al. [32]. Subsequently, a total bed volume (bv) of 50 mL was obtained in each tube.

During the first series of tests, two EDDS solution molarities ([EDDS]) were tested (0.36 and 3.6 mM). The flushing solution was leached through the columns using IVEK rotary pumps (IVEK Corporation, North Springfield, Vermont) at three different percolation velocities (1.7, 2.1, and 2.7 mL·h^−1^), which corresponded to three Empty-Bed Contact Times (EBCTs), i.e., 33, 27, and 21 h. The total treatment lasted 20 days. The solution collected at the bottom of the column was sampled at different times of the test and analyzed to measure the concentration of the following metals: Cu, Zn, Fe and Mn. 

In order to evaluate metals mobility in the soil column, other tests were performed for selected experimental conditions ([EDDS] = 3.6 mM and EBCT = 33 h). After the flushing process, the soil column was divided into four sections, and the metal concentration was measured in each of them (Soil Flushing Test 1, SF1). Furthermore, sequential extraction was carried out on the soil contained in each layer to evaluate the changes of PTEs fractionation after the treatment. A comparative test using only pure water as a flushing agent was also performed. A second series of tests (Soil Flushing Test 2, SF2) was performed to evaluate metal transportation along the columns. For this purpose, before the flushing process, the columns were divided into four layers, each of them containing 10 g of the contaminated soil. The flushing solution was sampled at the bottom of each layer and analyzed for PTEs and main cations concentration. Flushing operative conditions were the same as also adopted for the previous test. Finally, a third series of tests (Soil Flushing Test 3, SF3) was conducted to study the release of PTEs in the leachate after the soil flushing treatment (i.e., release due to the rain phenomenon). In this case, only 4 bv of the EDDS solution was injected through the columns at the beginning of the test, and then only pure water was added as a flushing agent. The flushing solutions were collected at the bottom of the columns and analyzed for PTEs evaluation. Also in this case, flushing operative conditions set for SF1 and SF2 were adopted.

### 2.4. Soil Washing Lab Scale Tests

Comparative soil washing tests were conducted on the same soil, at the lab scale, in 50-mL plastic reactors. Two different values of the liquid to solid ratio (LSR) were chosen, namely 5:1 and 10:1 (*v*/*w*). Soil washing parameters were optimized on the basis of the results of previous studies [24,33] conducted on the same soil. In particular, an extracting solution of [EDDS] = 0.36 mM was adopted to study the process efficiency in EDDS deficiency conditions, whereas an extracting solution of [EDDS] = 3.6 mM was used to achieve the best process performance. Two reaction times (i.e., 48 and 96 h) were selected since a previous work [33] proved these times to be efficient for achieving the PTEs equilibrium conditions. All tests were conducted in triplicate to reduce experimental errors. The metal concentration in the exhausted solution was measured by atomic adsorption spectrometry following the procedure described in Section 2.2. One-way and two-way analysis of variance (ANOVA) were used to analyze the statistical differences among treatments. Comparisons were made with the post-hoc Tukey’s Honestly Significant Difference HSD test. Statistical significance was assumed at *p* < 0.05. Statistical analyses were conducted in Microsoft^®^ Excel 2013/XLSTAT©-Pro (Version 7.2, 2003, Addinsoft, Inc., Brooklyn, NY, USA) and GraphPad Prism 6.0 (GraphPad Software, San Diego, CA, USA).

## 3. Results and Discussion

### 3.1. Soil Characterization 

The initial pH of the soil was close to neutral value (7.21) and the LOI index was 7.01%. The study of the particle size distribution resulted in classifying the soil as a loam, confirming its suitability for plant growth and agricultural activities. Among all of the measured PTEs, only Cu and Zn exceeded the threshold values (TVs) established by the European Regulation (Table 1) [34]. 

The PTEs reported in Table 1 are very common soil pollutants as they can be found in some pesticides and fertilizers [35]. High concentrations of Cu and Zn can represent a serious risk for human health since both metals could become toxic [36], making soil treatment necessary. Results of the sequential extraction showed that the percentage of Cu and Zn in the bioavailable fraction (sum of the first three fractions) was 81% and 70%, respectively (Figure 5). These values further confirmed the risk of Cu and Zn migration from the soil to agricultural products.

### 3.2. Optimization of the Soil Flushing Process

**Figure 2:**
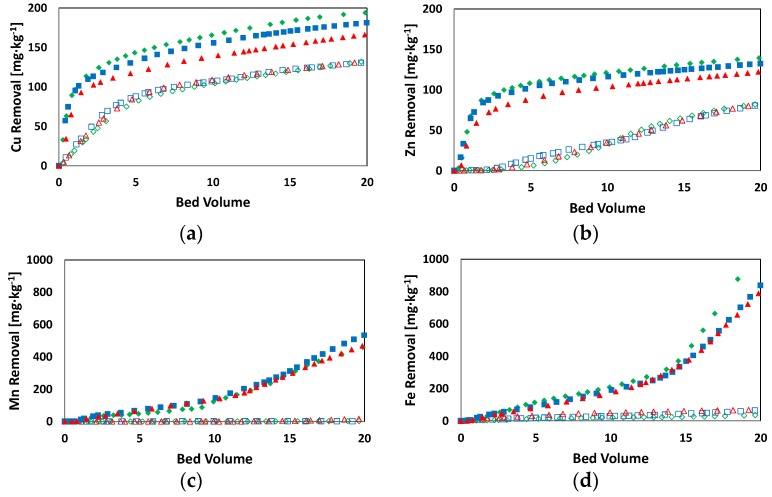
Breakthrough curves at different Empty-Bed Contact Times (EBCTs) with respect to bed volume. [EDDS] = 3.6 mM: 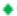 EBCT = 33 h; 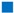 EBCT = 27 h; 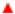 EBCT = 21 h; [EDDS] = 0.36 mM: 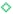 EBCT = 33 h; 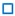 EBCT = 27 h; 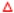 EBCT = 21 h—(**a**) Cu; (**b**) Zn; (**c**) Mn; (**d**) Fe.

**Figure 3:**
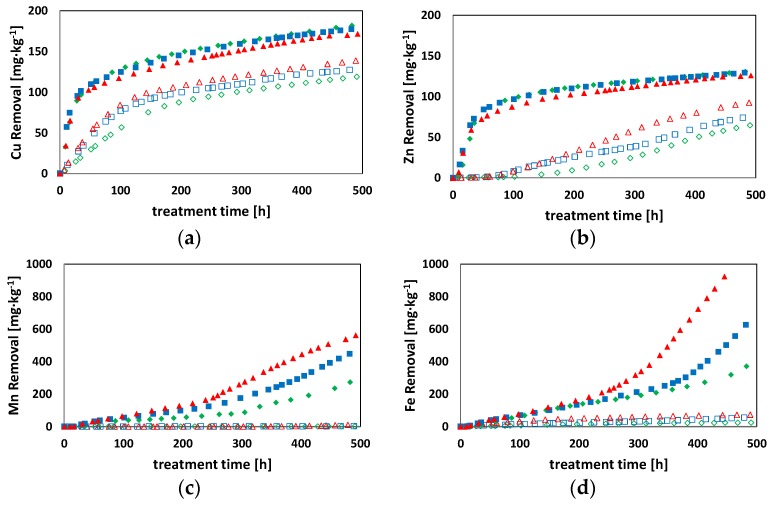
Breakthrough curves at different EBCTs with respect to treatment time. [EDDS] = 3.6 mM: 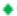 EBCT = 33 h; 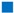 EBCT = 27 h; 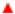 EBCT = 21 h; [EDDS] = 0.36 mM: 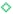 EBCT = 33 h; 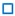 EBCT = 27 h; 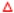 EBCT = 21 h—(**a**) Cu; (**b**) Zn; (**c**) Mn; (**d**) Fe.

Figure 2 and Figure 3 report the results of Cu, Fe, Mn, and Zn removal efficiency obtained during the flushing tests. Results from Figure 2 displayed that the EBCT influenced the flushing process only at high EDDS concentrations, resulting in a different removal efficiency. These differences were accentuated mainly for the lower values of bv (Figure 2). Actually, the slopes of the curves related to Cu and Zn removal were equal when the bv was higher than 2. On the contrary, no effects on the removal efficiency at different treatment times were found for tests characterized by significantly high EDDS concentrations (Figure 3). In this case, in fact, it was possible to achieve significant PTEs removal efficiency for all of the investigated treatment times. At a high EDDS concentration (3.6 mM), Cu and Zn extraction rates were characterized by a fast kinetic only in the first period of the treatment, whereas in the remaining time the kinetics were significantly slower. This observed effect was likely due to the following two phenomena: (i) an immediate extraction of the two metals from the carbonate fraction where they are in the di-valent form, i.e., Cu^2+^ and Zn^2+^; (ii) a subsequent oxidation of the metals from the mono- and zero- to the di-valent form, as a consequence of the humic acid reduction [33,37]. Actually, metals in zero-valent form cannot be complexed as Me-EDDS [33]. On the contrary, the extraction rates of Fe and Mn increased during the overall treatment time. In detail, a lower removal was observed at the beginning of the flushing treatment followed by a gradual increase at the end of the process. Such a result was attributed to the higher affinity of EDDS for Cu and Zn compared to Fe and Mn, which mainly affected the removal efficiencies at the beginning of the flushing treatment. Subsequently, the effect of the high concentration of EDDS prevailed on the previous factors, resulting in a relevant Fe and Mn removal efficiency. Moreover, the pattern was also related to the occurrence of different metals mobilization depending on the redox potential conditions of the soil. In fact, it is generally reported that Cu and Zn have a higher mobility rate under oxidizing conditions while Fe and Mn are characterized by higher mobility at lower values of soil oxidizing potential [38]. During the tests, after the first few days the soil in the column became water-saturated. This caused a reduction of the oxidizing potential and the conversion of Fe and Mn to their more leachable reduced forms [39].

During tests conducted with [EDDS] = 0.36 mM solution at different EBCTs, the extraction process was not influenced by the different values of bv. Results in Figure 2, in fact, show an overlap of the extraction curves. 

This result was due to the occurrence of a total EDDS complexation with metals, as a consequence of the low concentration of EDDS in the solution. Hence, the EDDS concentration represented a limiting factor of the treatment velocity. As the EDDS was fully complexed, the metal exchange mechanisms acquired a higher importance in the process. Indeed, in all tests with lower EDDS concentration (i.e., [EDDS] = 0.36 mM), Cu was the only metal with an excellent initial extraction rate. Results showed that for bv values up to 4 the amount of leached Cu was 77 mg·kg^−1^, corresponding to approximately 49 ± 5 µmol (Figure 2). This value was higher than 70% of the corresponding EDDS moles injected (72 µmol). Such a result could be ascribable to the adsorption of a small amount of EDDS onto the soil and the formation of Cu-EDDS complexes with the remaining EDDS [40]. Then, Zn-EDDS complexes started to form only for bv values higher than 4, as a result of the different values of the stability constant (Kst) of the two PTEs-EDDS. In fact, the Kst of Cu-EDDS is higher than that of Zn-EDDS [41]. For fixed values of treatment time, the EBCT affected the extraction rate of metals since lower EBCT values corresponded to a higher amount of the injected EDDS solution, and resulted in higher metal extraction rates (Figure 3). As the EDDS was totally complexed by Cu and Zn, the cations Fe and Mn could not form complexes with the flushing agent, and therefore could not be extracted. The pH and the LOI index of the soil were evaluated at the end of the tests. The soil pH displayed a value equal to 7.05 ± 0.2, assessing for the investigated soil buffering capacity in minimizing pH changes [42]. Likewise, the LOI value was observed at the end of the test confirming no soil characteristic alteration due to the EDDS involvement.

### 3.3. Soil Washing Process and Comparison of the Removal Efficiencies

**Figure 4:**
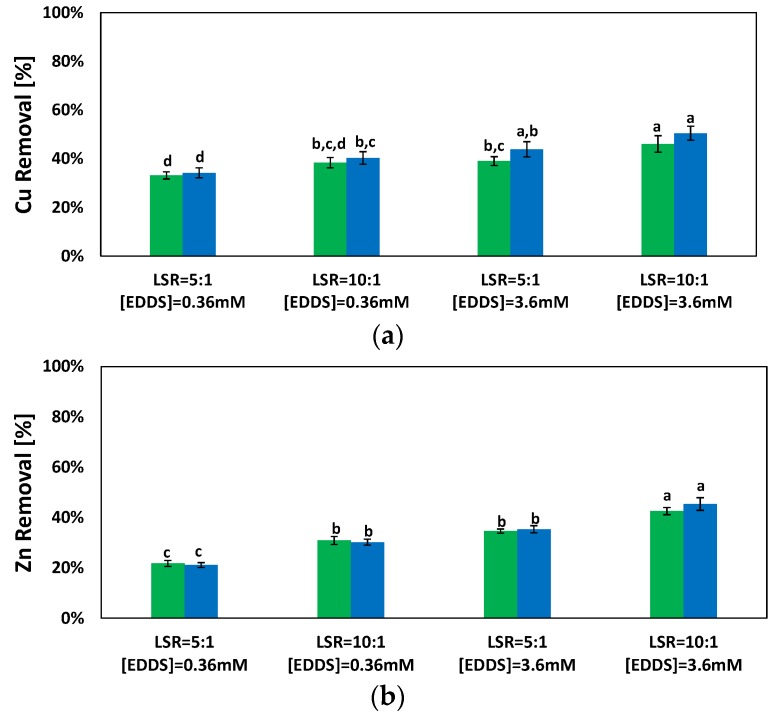
Cu and Zn removal by soil washing treatment at different times. 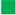 48 h; 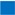 96 h—(**a**) Cu; (**b**) Zn.

The results obtained from the soil washing tests, with constant LSR and different EDDS concentrations, showed different removal trends for Cu and Zn (Figure 4). In all tests, a substantial increase in the removal efficiency occurred in the first 48 h, followed by a non-significant increase in the removal efficiency at 96 h. This was also confirmed by statistical analysis since no statistically significant differences between the removal efficiency at 48 and 96 h were observed (*p* > 0.05). These results were in accordance with previous investigations [33,43].

Results of tests conducted with varying the LSR showed significant differences in terms of the removal efficiency, especially for Zn, as also observed from the statistically significant differences (*p* < 0.05). It is worth noting that higher LSR values, achieved by increasing the liquid phase and keeping constant the EDDS solution molarity, induced a consequent increase of EDDS moles in the solution. Such a trend may be attributed to the following processes: (i) the presence of a certain amount of free EDDS non-complexed with metals; and (ii) the occurrence of metal exchange phenomena among the PTEs-EDDS complexes, which promotes the formation of Cu-EDDS or Zn-EDDS complexes [44]. 

In agreement with Tsang et al. [45], tests conducted with an EDDS concentration deficiency led to Fe and Mn removal efficiencies lower than 1%. At higher EDDS concentration, Fe and Mn removals slightly increased, but the overall removed amount of these elements was negligible compared to the initial total amount in the soil [46].

Comparing the results of the soil flushing and soil washing tests, it could be concluded that a significantly higher Cu removal efficiency was obtained with the soil flushing treatment considering the same amount of EDDS solution used. On the contrary, only a slightly higher Cu removal efficiency was achieved with the soil flushing treatment compared to the soil washing when the same treatment time was considered (Figure S1). 

### 3.4. Fractionation of PTEs/Main Cations in Different Soil Layers after Leaching

**Figure 5:**
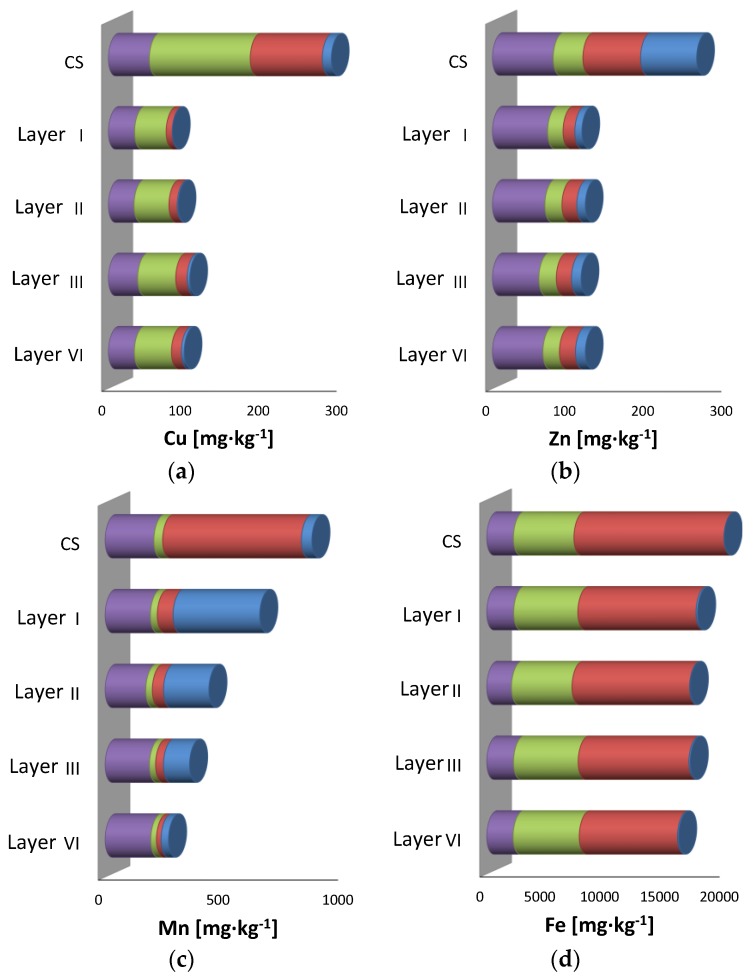
Values of metals in the four steps of the sequential extraction on the contaminated soil (CS) and on the four layers of the treated soil before and after SF1 tests. 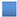 Exchangeable and weak acid soluble fraction, 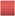 Reducible fraction, 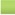 Oxidizable fraction; 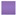 Residual Fraction (**a**) Cu; (**b**) Zn; (**c**) Mn; (**d**) Fe.

Interesting results were obtained from the sequential extraction procedure by comparing pre- and post-treatment metal distributions (Figure 5). Cu was initially bound to the organic substance and metal oxides/hydroxides complexes, while the amount of ions in the cation exchange sites was not relevant. A higher removal efficiency was observed for the Cu fraction bound to the organic substance and absorbed onto the metal oxides, as shown by the values of Cu extracted in the second and third steps. As regards Zn, instead, the highest removal efficiency was observed in the exchangeable and reducible fractions (steps 1 and 2). Small amounts of Cu and Zn were still found in the exchangeable and weak acid soluble fractions (step 1) after the treatment. This latter result was attributed to the EDDS amount adsorbed onto the soils after leaching [40,47].

Finally, Fe was extracted almost exclusively from the reducible fraction (step 2), while Mn was mobilized mainly from the reducible fraction (step 2) and partially deposited on the cation exchange sites along the soil column. The results showed (Figure 5) that the Cu and Zn concentrations at the end of treatment were lower than the threshold levels (Table 1) and their removal occurred mainly from the bioavailable fraction. This treatment displayed interesting outcomes since it allowed the reduction of the leaching of PTEs. In fact, the main removal occurred from the acid soluble fraction of the soil, which is generally characterized by fast metals mobilization [48] and represents a serious contamination risk for the environment [49].

### 3.5. Metals Transportation through the Columns

**Figure 6:**
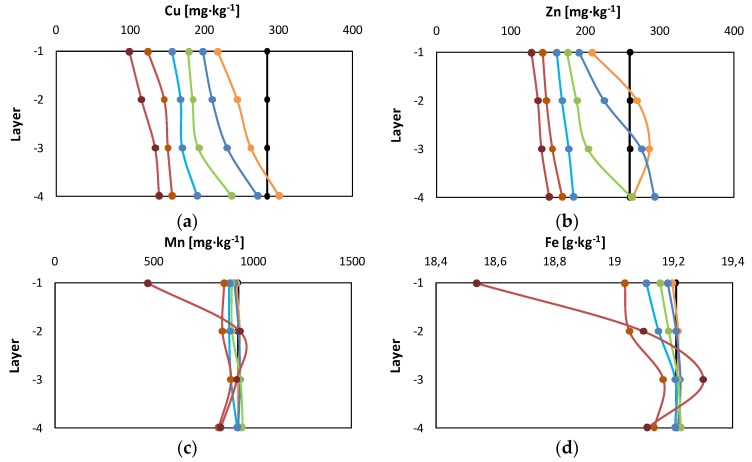
Cu, Zn, Mn, and Fe transport across soil column layers with respect to different values of bv: 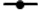 0 bv; 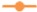 0.25 bv; 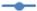 0.5 bv; 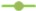 1 bv; 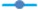 2 bv; 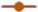 5 bv; 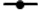 19 bv—(**a**) Cu; (**b**) Zn; (**c**) Mn; (**d**) Fe.

Figure 6 reported the results of metal profiles at different column layers obtained from SF2. Data were plotted at different bv values corresponding to different amounts of injected flushing solution. Metal concentration in the soil was evaluated by mass balance through the measure of their concentration in the spent solutions sampled at the bottom of each layer. 

The various metals exhibited different behaviors due to the occurrence of metal exchange phenomena or metal-EDDS complex adsorption. These phenomena were more significant for Zn than Cu.

The Cu concentration initially increased with the depth, therefore the concentration measured in the deepest layer was higher than that in the top layer. Nonetheless, after the injection of the first 0.25 bv of the flushing solution, the gradient tended to be gradually less pronounced, along with the reduction of the residual concentration in each layer. The same trend was observed for Zn only after the injection of the first 0.5 bv of the flushing solution. For this bv value, the distribution of Zn concentration with the depth was not monotonic at the beginning of the treatment, due to Zn release from the top layer and its adsorption in the following two layers. Upon increasing the bv values, the removal of Zn also occurred in the second layer but at a lower extent than in the top layer, and the released metal amount was adsorbed in the two successive layers. Finally, Zn removal also occurred in the third layer after 1 bv, and in the last layer after 2 bv injection. At the end, the Zn distribution was uniform, as was the Cu distribution. 

The obtained distribution of Cu with the depth was in contrast with the findings of Hauser et al. [21]. This result was ascribable to the different operative conditions set for the flushing tests. In the work of Hauser et al. [21], leaching tests were carried out with a non-continuous flow rate and non-water saturated soil. In such conditions the reduction from Cu^2+^ to elemental Cu occurred and the complex with EDDS did not form [33]. 

On the other hand, the distribution of Zn with respect to the depth was similar to that obtained in previous studies [21,50]. This result was attributed to the non-occurrence of Zn^2+^ reduction to elemental Zn. In fact, it is reported [50] that Zn can be released as Zn^2+^ and Zn(OH)^+^ according to the following reactions (r_1_–r_2_):

r_1_) Zn^2+^ + HEDDS^3−^ ↔ ZnEDDS^2−^ + H^+^
r_2_) Zn(OH)^+^ + HEDDS^3−^ ↔ ZnEDDS^2−^ + H_2_O

and H^+^ formation can cause ZnO dissolution (r_3_–r_3_):

r_3_) ZnO + 2H^+^ ↔ Zn^2+^ + H_2_O
r_4_) ZnO + H^+^ ↔ ZnOH^+^

Moreover, Zn oxy-hydroxides can be chelated from EDDS as follows (r_5_):

r_5_) ZnO + HEDDS^3−^ ↔ ZnEDDS^2−^ + OH^−^.

As regards Fe and Mn distribution throughout the column depth, it was confirmed that the removal took place starting from 2 bv. A uniform removal profile was observed along the depth during the first period of the treatment. After 10 bv a relevant removal of Fe and Mn occurred in the top layer in addition to the release of the metals from the successive layers. However, the amount of Fe and Mn removed was negligible compared to their initial concentration in the soil. This confirmed that pollutants can be removed without damaging the soil if an appropriate technique is selected.

### 3.6. Release of PTEs in the Water after the Treatment

**Figure 7:**
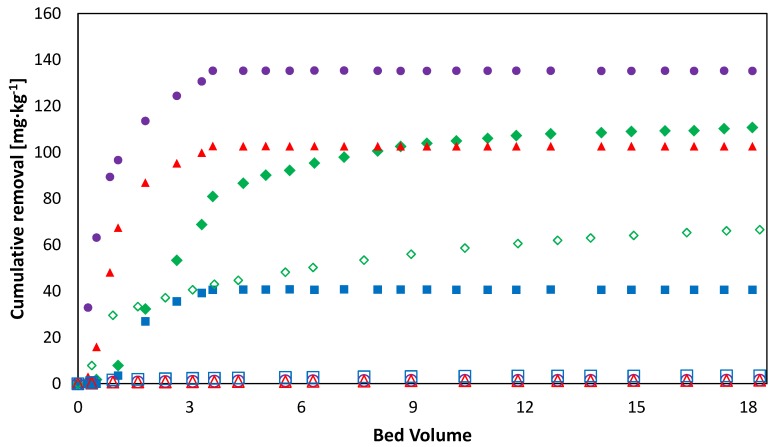
Cu, Zn, Fe, and Zn cumulative removal from the soil as a function of bv, during the SF3 tests. Full symbols: 0–4 bv [EDDS] = 3.6 mM, 4–18 bv [EDDS] = 0 mM—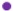 Cu; 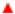 Zn; 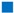 Mn; 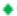 Fe. Empty symbols: 0–18 bv [EDDS] = 0 mM. 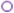 Cu; 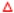 Zn; 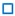 Mn; 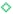 Fe.

Results from the SF3 tests are reported in Figure 7. As can be seen, there was no release of Cu, Zn, or Mn in the water once the injection of EDDS was stopped (i.e., after 4 bv of solution injection), confirming the effectiveness of the treatment. This indicates that the PTEs were likely bound in forms that exhibit a low potential release in the environment and low bioavailability for the living organisms [51]. The only exception was represented by the first 0.5 bv of removal (4–4.5 bv), characterized by the presence of a low metals concentration, due to the retention of a certain amount of EDDS in the column. This occurrence led to the recommendation of soil flushing with water as a final step of the remediation treatment. Conversely, the release of Fe persisted all along the treatment until the same removal efficiency was achieved, as observed in the control test.

## 4. Conclusions

The present study assessed the feasibility of performing soil flushing treatment with EDDS solution for remediating natural soils contaminated by Cu and Zn. An almost total removal of the bio-available fractions of these metals was achieved. Although the main factor that influenced the metals removal efficiency was the EDDS concentration in the extracting solution, this study proved that the effectiveness of the soil flushing was also dependent on several other factors, including contaminated soil depth as well as the different PTEs-EDDS complexes affinity. Moreover, tests under EDDS-deficiency conditions have further demonstrated that the Cu and Zn removal efficiency was lower due to their re-adsorption onto the soil and/or because of the occurrence of metal exchange processes. In order to take into account all of these aspects, it is essential to carry out bench-scale tests before performing a full-scale soil remediation treatment. Finally, the study proved that soil flushing, besides having a lower environmental impact and lower operative costs compared to soil washing (e.g., no soil excavation is required), is capable of achieving the same high removal efficiency, producing a smaller volume of contaminated spent solution and consuming a minor amount of chemical agents.

## Figures and Tables

**Table 1:** Soil characterization.

PTEs	Cd	Cr	Cu	Fe	Mn	Ni	Pb	Zn
Soil (mg·kg^−1^)	0.13 ± 0.01	23.0 ± 1.2	296 ± 10	19842 ± 1230	913.9 ± 31.1	11.9 ± 2.1	21.9 ± 0.2	277 ± 3.0
Threshold values (mg·kg^−1^) [34]	1	100	100	-	-	50	60	200

## Supplementary Materials

The following are available online at http://www.mdpi.com/1660-4601/15/3/543/s1, Figure S1: Cu and Zn removal by soil flushing treatment at different times.  48 h;  96 h and at different values of bv—(a) Cu 4 bv; (b) Zn 4bv; (c) Cu 8 bv; (d) Zn 8 bv.
