# Supplementary material for: Ethylenediamine-N,N′-Disuccinic Acid (EDDS)—Enhanced Flushing Optimization for Contaminated Agricultural Soil Remediation and Assessment of Prospective Cu and Zn Transport
Source: Int J Environ Res Public Health. 2018 Mar 18;15(3):543. doi: 10.3390/ijerph15030543 (PMC5877088; doi:10.3390/ijerph15030543)
Supplement: Supplementary file 1 [file ijerph-15-00543-s001.pdf]

Supplementary Material

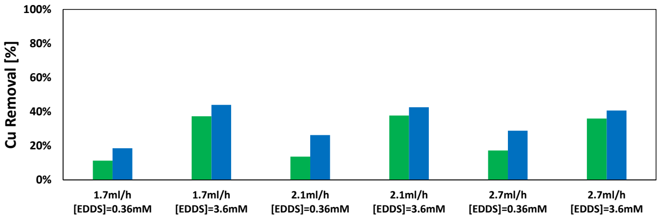

(**a**)

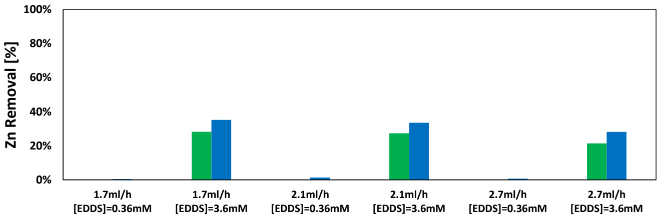

(**b**)

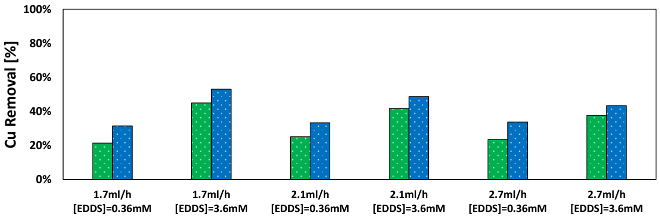

(**c**)

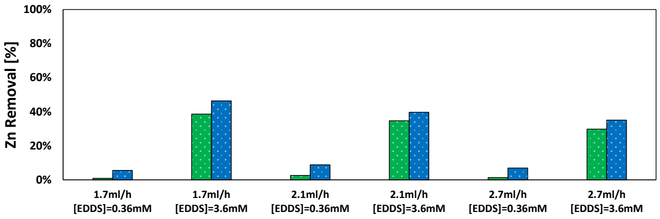

(**d**)

Figure S1. Cu and Zn removal by soil flushing treatment at different times. .) 48 h;) 96 h and at different bv – a) Cu 4 bv; b) Zn 4bv; c) Cu 8 bv; d) Zn 8 bv.
